# HyperVein: A Hyperspectral Image Dataset for Human Vein Detection

**DOI:** 10.3390/s24041118

**Published:** 2024-02-08

**Authors:** Henry Ndu, Akbar Sheikh-Akbari, Jiamei Deng, Iosif Mporas

**Affiliations:** 1 School of Built Environment, Engineering and Computing, Leeds Beckett University, Leeds LS1 3HE, UK; h.ndu6387@student.leedsbeckett.ac.uk (H.N.);; 2 Department of Engineering and Technology, School of Physics, Engineering & Computer Science, University of Hertfordshire, Hatfield AL10 9AB, UK

**Keywords:** hyperspectral imaging, vein detection, image classification

## Abstract

HyperSpectral Imaging (HSI) plays a pivotal role in various fields, including medical diagnostics, where precise human vein detection is crucial. HyperSpectral (HS) image data are very large and can cause computational complexities. Dimensionality reduction techniques are often employed to streamline HS image data processing. This paper presents a HS image dataset encompassing left- and right-hand images captured from 100 subjects with varying skin tones. The dataset was annotated using anatomical data to represent vein and non-vein areas within the images. This dataset is utilised to explore the effectiveness of dimensionality reduction techniques, namely: Principal Component Analysis (PCA), Folded PCA (FPCA), and Ward’s Linkage Strategy using Mutual Information (WaLuMI) for vein detection. To generate experimental results, the HS image dataset was divided into train and test datasets. Optimum performing parameters for each of the dimensionality reduction techniques in conjunction with the Support Vector Machine (SVM) binary classification were determined using the Training dataset. The performance of the three dimensionality reduction-based vein detection methods was then assessed and compared using the test image dataset. Results show that the FPCA-based method outperforms the other two methods in terms of accuracy. For visualization purposes, the classification prediction image for each technique is post-processed using morphological operators, and results show the significant potential of HS imaging in vein detection.

## 1. Introduction

Vein detection plays a critical role in the medical field, as numerous surgical procedures rely on accessing the vascular system, necessitating accurate identification and localization of veins within the human body [1,2,3,4,5]. Medical practitioners often find it difficult to precisely locate veins in the human body [2,4]. This issue is particularly prevalent in certain patient populations, including children, individuals with excessive subcutaneous fat, and patients with darker skin tones [1,2,4]. When veins are inadequately visible, medical professionals are compelled to rely on their anatomical knowledge to perform blind sticks during medical procedures. Relying solely on a practitioner’s skills and anatomical knowledge can result in imprecise outcomes [1]. This makes precise vein detection essential in modern medical practice. Failed venipuncture attempts can lead to complications such as vein thrombosis [6], hematoma, or nerve injuries, potentially causing conditions like “causalgia” or complex regional pain syndrome (CRPS) [3,7]. Moreover, accurate vein detection is vital for studying and managing cancer, as it provides valuable insights into the anatomical relationship between arteries and veins in tumours [5,8,9].

Improving vein detection methods can significantly enhance patient care and treatment outcomes. Currently, a variety of devices have been developed to aid healthcare workers in locating subcutaneous veins of patients for delivering intravenous or surgical treatments. These devices utilize different techniques such as trans-illumination, photo-acoustic, ultrasound, and Near-Infrared (NIR) imaging to aid in visualizing non-visible veins of patients. Each technique possesses distinct advantages and drawbacks, but NIR imaging has emerged as particularly suitable for vein localization during intravenous treatments [1,10,11,12]. By employing non-ionizing light rays, NIR imaging can penetrate deep within skin tissues to acquire clear images of the venous structure. However, despite the advancements in NIR imaging, challenges persist in accurately and reliably detecting veins, especially in complex surgical scenarios. To address this gap, Hyper-Spectral Imaging (HSI) offers a promising solution.

HSI captures spectral radiation across the visible to near-infrared electromagnetic spectrum, generating distinct images for each spectral band. It captures hundreds of continuous spectral bands, forming a datacube often referred to as a hypercube. This comprehensive data representation enables the acquisition of detailed information beyond what the human eye can perceive, providing valuable insights for various applications, e.g., agriculture, environmental monitoring, geology and mineral exploration, and medical imaging [13,14]. Widely explored in remote sensing applications, HSI offers a powerful tool for analysing and interpreting complex data from a diverse range of sources. Despite its successes in the medical field, HSI for human vein detection is yet to be investigated. The capabilities of HSI to capture rich spectral information may provide reliable data for vein detection, enabling precise vein localization during surgeries and other medical procedures.

To address the challenges in accurate vein detection, a hyperspectral (HS) image dataset is presented. This dataset stands out for its diverse representation of skin tones, inclusion of left and right hands from 100 subjects, and meticulous annotation to map out veins and the surrounding hand areas. The HS image dataset serves as a crucial contribution to the field, providing a rich resource for evaluating and advancing vein detection methodologies in real-world scenarios.

HSI data can be quite large, posing challenges in terms of manageability and demanding high computational resources. Consequently, these factors can potentially impact vein detection performances and, consequently, overall classification accuracy. Therefore, dimensionality reduction techniques are commonly employed to reduce their complexity. The selection of the most appropriate dimensionality reduction technique depends on the specific application’s requirements and the technique’s performance in accurately preserving essential vein detection features while reducing data complexity. For this research, three dimensionality reduction techniques that have previously been successfully used in HS data analysis, namely Principal Component Analysis (PCA), Folded Principal Component Analysis (FPCA), and Ward’s Linkage Strategy using Mutual Information (WaLuMI) were chosen for experimentation.

HSI has made significant contributions to the medical field, with a diverse range of applications. One such application involves the calculation of tissue oxygen saturation [15], offering valuable insights into oxygen levels within tissues. It has also been effectively employed to monitor relative spatial changes in retinal oxygen saturation [16], providing detailed observations of oxygen variations in the retinal region. Additionally, this imaging technique has been used to obtain the optimum range of illumination for venous imaging systems [1].

This paper makes a substantial contribution to the field by introducing a curated HS image dataset of 100 subjects that are labeled to map out the vein and the rest area of the hand, forming ground truth images. The dataset covers a wide range of skin tones from diverse ethnicities. This dataset is then used to study the effectiveness of PCA, FPCA, and WaLuMI in conjunction with the Support Vector Machine (SVM) binary classifier for vein detection. The annotated HS image dataset allows evaluation of the performance of each of the dimensionality reduction methods in the context of real-world vein detection tasks. By leveraging these dimensionality reduction techniques, salient features are extracted from the HS data, enabling the vein detection algorithm to identify vein patterns accurately. The rest of this paper is organized as follows: Section 2 describes the HS image data acquisition method. Section 3 discusses the vein detection methodology and Section 4 provides insights into the experiments conducted and the results obtained. Finally, Section 5 draws the conclusion.

## 2. Materials and Method

### 2.1. Hyperspectral Image Acquisition

**Figure 1:**
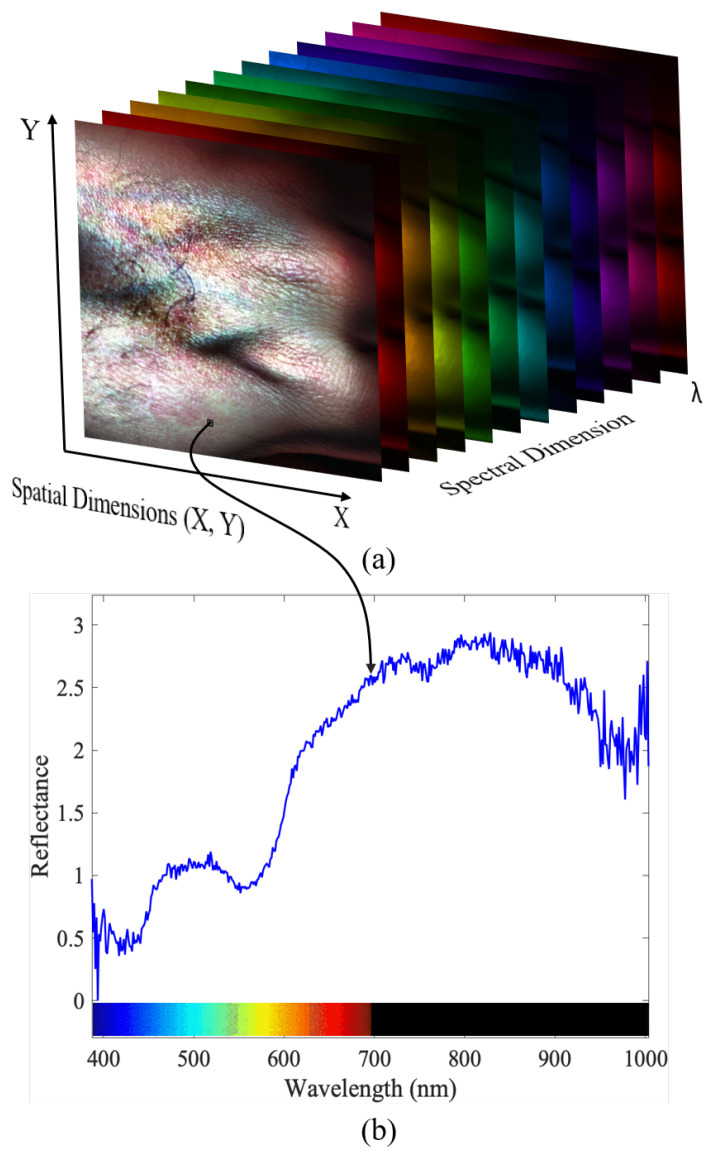
A hyperspectral image: (**a**) A schematic view of a hyperspectral image of human hand. (**b**) The spectral graph of the spectrum of a pixel from the hand. The graph represents the reflectance values for each wavelength captured by the pixel.

To capture the HyperSpectral (HS) image data, the benchtop HSI system manufactured by Resonon Inc. (Bozeman, MT, USA) was used. The Resonon’s benchtop HSI system comprises of a Pika XC2 HSI camera, objective lens, linear translation stage, mounting tower, halogen line light with stabilized power supply, a calibration tile, and a system with Spectronon software pre-loaded. The Pika XC2 camera has a spectral range of 400–1000 nm (nanometer), spectral resolution of 1.9 nm, spectral channels of 447, spatial pixels of 1600, and spectral bandwidth of 1.3 nm. Every pixel within the HS image contains a series of reflectance values across various spectral wavelengths, revealing the spectral signature of that particular pixel. Figure 1 illustrates a schematic depiction of a sample captured HS image.

**Figure 2:**
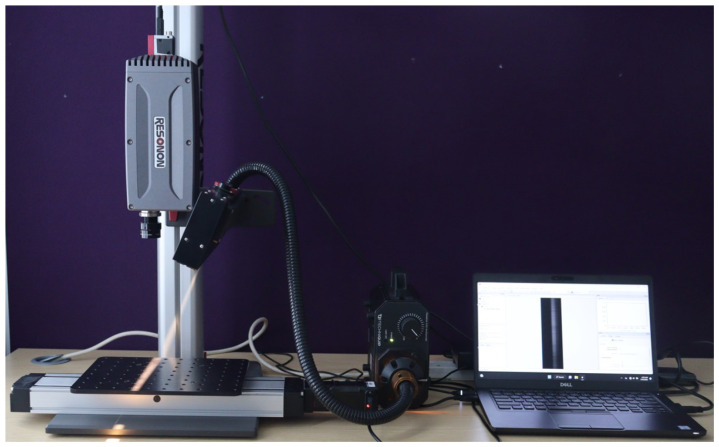
Hyperspectral image data acquisition setup.

To setup the HS data acquisition system, the camera is mounted on the tower directly above the motorized linear translation stage. The lighting assembly is positioned and secured on the tower to illuminate in the direction of the stage baseplate from above. A halogen line light, as the light source, provides stabilized broad-band illumination on the human hand to be captured. To optimize the setup and improve data acquisition capabilities, the camera and lighting were carefully adjusted along the length of the tower. Figure 2 shows the HS image data acquisition setup.

**Figure 3:**
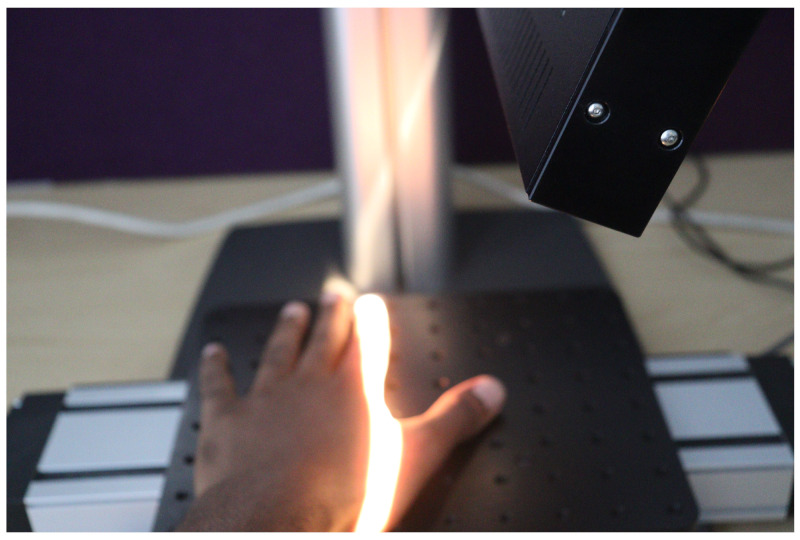
A human hand being captured using the push-broom technique.

To initiate data capturing, the camera underwent calibration to ensure precise measurements. Throughout the data acquisition process, a consistent distance was maintained between the camera lens and the stage baseplate. During data acquisition, the linear translation stage moves, causing the hand to be translated beneath the camera. The HS camera utilizes the push-broom technique for imaging. This technique involves the camera scanning the object line by line using its inbuilt tunable filters or liquid crystal filters. By electronically adjusting these filters, the camera captures different spectral wavelengths of light. As the linear translation stage moves along the scanning direction, the camera sequentially captures HS information from different parts of the object. This allows the construction of spectral intensity images for each wavelength, resulting in a comprehensive HS image set. Figure 3 shows a human hand being captured.

The Spectronon software facilitates the visualization of the captured HS images and enables a comprehensive suite of intuitive hypercube analysis functionalities. Additionally, it offers control over the linear translation stage, allowing precise manipulation of the stage position for enhanced data acquisition. The hypercubes are captured in Band-Interleaved-by-Line (BIL) format, accompanied by the generation of a corresponding HeaDeR File (HDR) for each completed capture. The HDR file contains essential metadata that describes various aspects of the captured data.

### 2.2. HS Image Dataset

The capturing processes formed a HS image dataset comprising the left- and right-hand images of 100 participants meticulously captured, yielding a comprehensive collection of 200 images. The volunteer participants are a diverse group of individuals from various countries, including Asia, Africa, America, Britain, and Malaysia. The dataset encompassed individuals spanning different age groups and exhibiting distinct skin tones, representing a broad range of ethnicities for the experiments. To characterize the dataset, skin tone distribution is categorized according to the Fitzpatrick Scale [17]. The Fitzpatrick Scale was developed by dermatologist Thomas B. Fitzpatrick to classify skin color and response to ultraviolet radiation [18]. This scale serves diverse applications, including assessing skin cancer risk, guiding aestheticians in determining optimal laser treatment parameters for procedures like hair removal or scar treatment, and evaluating the potential for premature skin aging due to sun exposure. The Fitzpatrick Scale classifies skin tones into Types I to VI, representing a range from the lightest to darkest. For reference, Type I corresponds to very light or pale skin, while Type VI represents very dark or deeply pigmented skin. The statistical summaries are presented in Table 1 and Table 2.

Table 1 outlines essential statistics regarding the dataset composition. Notably, the dataset consists of 200 hand images, representing both the left and right hands of the 100 participants. The gender distribution reveals that 76% are male, and 24% are female. Ethnicity distribution shows a diverse representation, with 32% African, 59% Asian, and 9% European participants. Age distribution spans multiple categories, with the majority falling within the 26–30 age group (28%). Furthermore, the majority of individuals in the dataset exhibit skin tones classified as Type III (Medium) and Type IV (Olive), each accounting for 21% and 22%, respectively.

Table 2 provides a detailed breakdown of skin tone distribution within each ethnic group, adhering to the Fitzpatrick Scale. Noteworthy findings include the prevalence of Type IV (Olive) and Type V (Brown) skin tones among African participants, constituting 8% and 13% of the group, respectively. In the European group, the majority exhibit Type I (Light) and Type II (White) skin tones, accounting for 6% and 2%, respectively. Asian participants exhibit a more balanced distribution across various skin tones, with Type II (White) and Type III (Medium) being the most prevalent.

**Figure 4:**
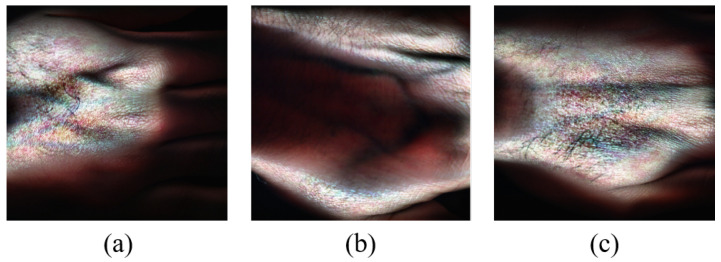
Sample RGB image representations of some of the HS hand images from the dataset: (**a**) Asian male, (**b**) Asian male, (**c**) African male. The RGBs are generated using three channels of the HS image.

**Figure 5:**
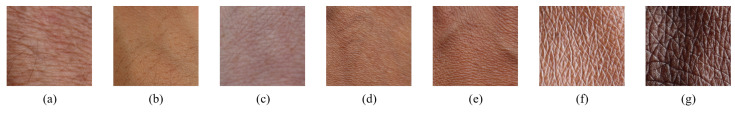
RGB images showing diversity of skin tones captured: (**a**) British male; (**b**) Asian male; (**c**) British female; (**d**) Asian male; (**e**) Indian male; (**f**) Asian male; (**g**) African male.

Figure 4 shows RGB image representations of some HS images from the dataset generated for visualization purposes using the hypercubes. This was achieved for each of the showcased HS images by selecting three specific channels from their hypercube and mapping them to the red, green, and blue channels of the RGB image. The captured hand images have spatial dimensions of 2800×1600 pixels and a spectral dimension of 462 bands. Some samples of the captured skin tones are shown in Figure 5.

### 2.3. Vein Detection Methodology Using HSI and Ground Truth

Vein detection using HSI is a vital process essential for medical applications. The procedure involves collecting a HS image dataset focused on anatomical regions like human hands and meticulously annotating ground truth images. These annotations, performed by experts, designate each pixel as part of the skin or vein, which can be represented as a binary class having 1 (vein) and 0 (skin). This ground truth is crucial for the subsequent training and evaluation of classifiers, particularly Support Vector Machines (SVM).

In the training phase, reduced-dimensional HS data, often obtained through techniques like PCA or FPCA, is fed into the SVM binary classifier. The classifier learns intricate patterns from the training dataset, associating spectral features with corresponding ground truth labels. During testing, the trained SVM is applied to new HS images, classifying each pixel as 0 or 1 based on learned patterns. Importantly, this step is referred to as “detection”, signifying the identification of veins within the HS data.

In this binary classification setup, a pixel assigned the value 0 represents skin, while a pixel labeled 1 indicates the presence of a vein. The binary representation simplifies the complexity of HS data, enabling a clear distinction between relevant anatomical structures. Ground truth, through its association of spectral patterns with known vein locations, ensures the reliability and precision of the vein detection algorithm. The interplay between HS imaging, ground truth annotations, and SVM classification forms a robust methodology for accurate and reliable human vein detection, with significant implications for medical applications.

### 2.4. Preprocessing

**Figure 6:**
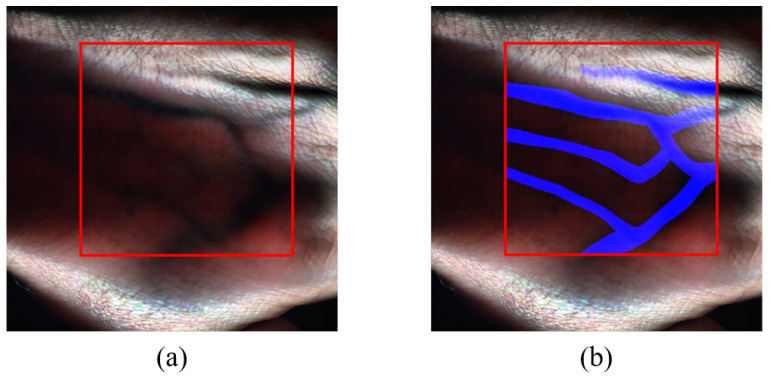
Sample HS image: (**a**) An RGB image representation with the Region Of Interest (ROI) highlighted in red. (**b**) Annotated RGB image, where veins are highlighted in blue.

The experimentations and data processing were conducted using MATLAB R2023a, a widely used software tool for scientific computing and data analysis, due to its comprehensive functionality and flexibility. The Region Of Interest (ROI) within the images has dimensions of 1024×1024×462 pixels. The ROI was carefully selected to encompass the essential spectral information relevant to the research objectives. The HS images were cropped to the size of the ROI. An estimated RGB image representation for each of the images’ ROI is then generated, which can be used for manual annotation. Figure 6a showcases the delineated ROI.

### 2.5. Data Annotation/Ground Truth Creation

To facilitate subsequent vein detection analysis, each HS hand image in the dataset was manually annotated to highlight the veins present in the hand. By doing so, ground truth images were created for each of the captured HS images as reference labels, and this was performed with the guidance of a medical expert and by using anatomical data to determine the vein locations in the images. The ground truth is a binary image with one representing the vein locations and zeros representing the rest. Figure 6b depicts the annotated RGB representation of the sample HS image data, with veins highlighted in blue.

## 3. Enhancement of Vein Detection Methodology

In this section, the methodology applied for enhanced vein detection in HS images is outlined, encompassing data pre-processing, dimensionality reduction, training and testing set separation, classification, performance assessment metrics, and visual representation of the classification outcome.

The methodology for human vein detection, employing the dataset of 200 HS images, introduces several novel elements that set it apart from existing methods in the field. In contrast to the work by Hamza et al. [20], which primarily focuses on blood vessel visualization in human skin using HSI, and that of Mzoughi et al. [21] exploring HS visualization for blood vessels with a focus on improving contrast ratios, the primary objective is the accurate detection of human veins for medical applications.

In the study by Hamza et al. [20], a technique for HS visualization of blood vessels in human skin is proposed. The experiment involves participants with diverse skin types, races, and nationalities, highlighting the adaptability of their approach. While the emphasis is on improving the first-attempt puncture success rate and reducing patient pain through enhanced blood vessel visualization, our proposed approach distinguishes itself by specifically targeting the challenging task of vein detection.

**Figure 7:**
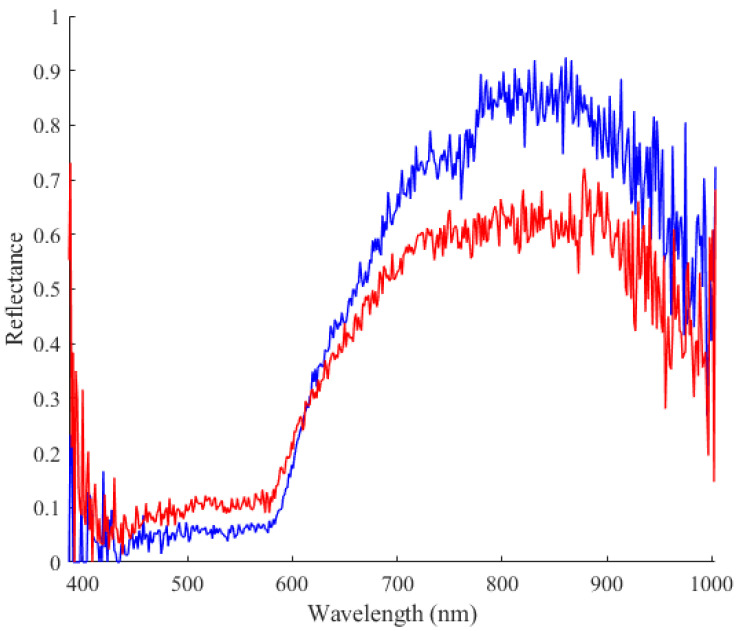
Spectral profiles depicting the reflectance spectrum of two distinct classes: skin (red) and vein (blue).

Furthermore, Mzoughi et al. [21] propose a technique for visualization of blood vessels using HS images, focusing on the improvement of contrast ratios. Their work involves an experiment with participants of different skin types, and they introduced new index formulae deduced through an exhaustive search. While their emphasis is on enhancing visualization and generating high-contrast blood vessel images for different skin types, our proposed approach goes beyond visualization, it addresses the crucial aspect of vein detection. See Figure 7.

Tailored to the intricacies of HS based vein detection, the methodology leverages a dataset of 200 HS images of human hands for training and testing. Significantly, ground truth annotations are meticulously crafted under the guidance of an expert in the field, ensuring accuracy and reliability in vein localization. In contrast to the aforementioned approaches, which primarily enhance visibility for general blood vessel localization, our proposed method specifically evaluates the effectiveness of dimensionality reduction techniques in conjunction with a Support Vector Machine (SVM) binary classifier. This unique combination allows the extraction of salient features from HS image data, facilitating accurate vein detection.

### 3.1. Dataset Preparation

Sixty (60)% of the HS images within the dataset were randomly selected and used for training purposes and the remaining images were used for testing and evaluation. This division allowed for the construction and assessment of the algorithm’s performance on unseen data.

To reduce the computation time during processing, while retaining essential information, the ROI of the images were cropped to 128×128×462 and likewise, their ground truth images were cropped to 128×128.

### 3.2. Dimensionality Reduction

The experiment involved three dimensionality reduction techniques, namely PCA, FPCA, and WaLuMI. The selection of these dimensionality reduction techniques for the experimentation was driven by their distinct characteristics and potential benefits in the context of HS data analysis. These techniques have demonstrated effectiveness in related fields and show promise for exploring their applicability in HS studies. Their concepts are explained below.

#### 3.2.1. PCA for HS Images

Principal Component Analysis (PCA) [22,23,24] is a widely used statistical technique for dimensionality reduction and data exploration in various fields [25]. It enables the analysis of complex datasets by transforming them into a new set of uncorrelated variables called principal components [26]. These components capture the maximum variance in the data, allowing for a simplified representation without significant loss of information. PCA has proven to be particularly valuable in numerous applications, including image processing, pattern recognition, and feature extraction.

In HSI, PCA has been successfully utilized for dimensionality reduction [24,27,28]. PCA’s ability to capture essential spectral variations and effectively reduce the dimensionality of HS data has led to its widespread adoption in this domain. HS images contain rich spectral information captured within a wide range of spectral bands. PCA aims to transform the original high-dimensional HS data into a new set of orthogonal axes called principal components. These components are ordered by the amount of variance they capture, with the first component capturing the highest variance, the second component capturing the second highest variance, and so on.

Mathematically, given a HS data set matrix *X*, where each row corresponds to a pixel and each column corresponds to a spectral band, PCA can be applied for data reduction and feature extraction of a HS image data as follows:

1. Mean-Centering: subtract the mean of each band from the corresponding column of *X* to center the data.
2. Covariance Matrix: calculate the covariance matrix by
  (1) $$C=\frac{1}{n-1}X^{T}X$$
  where *n* is the number of samples (pixels).
3. Eigen Decomposition: compute the eigenvectors and eigenvalues of the covariance matrix *C*. The eigenvectors form the principal components, and the eigenvalues represent the amount of variance captured by each component.
4. Data Projection: select the top *k* eigenvectors corresponding to the *k* highest eigenvalues to form a projection matrix *P*. Multiply the original data matrix *X* by *P* to obtain the lower-dimensional representation *Y*.

#### 3.2.2. FPCA for HS Images

Folded Principal Component Analysis (FPCA) [29] is an extension of PCA that takes into account the spatial information inherent in HS images. Unlike traditional PCA, which treats each pixel independently, FPCA considers the correlation between neighboring pixels. It leverages the interplay between spectral and spatial information to enhance dimensionality reduction and feature extraction.

In FPCA, the fundamental idea is to convert each spectral vector into a matrix format, enabling the direct calculation of a partial covariance matrix. This matrix is then accumulated for eigen-decomposition and data projection, effectively incorporating spatial relationships into the analysis.

FPCA can be implemented on HS data with important parameters *H* (fold size) and *W* (number of spectral bands in each segment) as follows:

1. Matrix Transformation: for each pixel’s spectral vector, a matrix is constructed where each row contains a segment of *W* spectral bands. The entire spectral signature, represented by *F* bands, is divided into *H* segments. This transformation allows for capturing spectral-spatial interactions within a local context.
2. Partial Covariance Matrix: a partial covariance matrix is computed directly from these segmented matrices. This matrix reflects the interactions between different spectral bands within each segment, encapsulating both spectral and spatial information.
3. Eigen Decomposition and Projection: the accumulated partial covariance matrices are subjected to eigen decomposition. The resulting eigenvectors represent directions of maximum variance within the folded spectral-spatial data. By selecting the top *k* eigenvectors associated with the largest eigenvalues, a projection matrix is formed.

When H=1, FPCA simplifies to conventional PCA, treating each pixel’s spectral vector individually. As *H* increases, spatial context is increasingly incorporated. A larger *H* enables capturing broader spatial interactions but creates increased computational complexity. FPCA has previously been successfully applied in HSI for efficient dimensionality reduction and feature extraction [28,29,30].

#### 3.2.3. WaLuMI for HS Images

Ward’s Linkage Strategy using Mutual Information (WaLuMI) [31] is a technique that combines hierarchical clustering using Ward’s linkage method [32] with mutual information as a similarity measure for HS image analysis. Hierarchical clustering groups pixels based on their similarity, creating a dendrogram that represents the hierarchy of pixel associations. Mutual Information (MI) is used as a criterion to measure the similarity between pixels. By utilizing mutual information and hierarchical clustering, WaLuMI considers both spectral and spatial information to discard redundant information in HS data, hence, leading to efficient data reduction in HS images. WaLuMI can be implemented on HS data as follows:

1. Mutual Information Calculation: compute the mutual information between spectral vectors of pixels. Mutual information measures the amount of information shared between two variables, indicating how much knowing one variable reduces uncertainty about the other.
  Let *I* be the input HS image with dimensions n×m, and *X* be the vectorized spectral data. The mutual information matrix is computed by
  (2) $$MI_{ij}=I\left(X_{i};X_{j}\right)$$
2. Wards Linkage: use the mutual information values to perform hierarchical clustering using the Wards linkage strategy. This strategy merges clusters that minimize the increase in the sum of squared differences within clusters.
3. Dendrogram Creation: as the algorithm progresses, a dendrogram is formed, representing the hierarchical structure of pixel groupings.

Each of these techniques was employed in a separate experiment. Initially, the HS images underwent dimensionality reduction using conventional PCA, after which a comprehensive analysis of the classification results was conducted.

Subsequently, the procedure was iterated by employing FPCA for dimensionality reduction, followed by a replication of the same process utilizing the WaLuMI technique. This systematic approach facilitated a thorough and comparative evaluation of how different dimensionality reduction techniques, namely PCA, FPCA, and WaLuMI, influenced the performance of vein detection.

### 3.3. Training and Testing Set Separation

The classifier was trained using 60% of the images of the HS image dataset and the rest of the images were used for testing to evaluate the classification performance for each technique. The training images were concatenated vertically to form the training data.

### 3.4. Classification: Support Vector Machine

Support Vector Machines (SVMs) [33] are widely used for classifying large data or handling noisy samples [5,34,35]. SVM has recently become a prominent method for HS image classification, gaining significant attention in the field [5,36,37,38]. Its popularity stems from its ability to find optimal decision boundaries that maximize the separation between different classes, even in complex data distributions [36]. By doing so, SVMs can effectively handle high-dimensional data and offer robust classification performance. SVM’s versatility and strong theoretical foundation have made it a valuable tool in various fields, including biomedical applications [5,39], pattern recognition [40], and data analysis [37,41].

Due to SVM’s successes in HSI applications, it was chosen to classify the HS data. The input for SVM classification consisted of the training data and its ground truth. Following training of the SVM classifier, it was applied to the testing images to predict the class labels of the test samples. By evaluating the classifier’s performance on unseen data, the effectiveness of the classification approach could be assessed. Throughout the SVM training phase, integration of a linear kernel function aided the classification process, resulting in a significant enhancement of vein detection performance in the experiment.

### 3.5. Performance Assessment Metrics

Following the classification stages, measures to evaluate the classification performance were implemented. This involved calculating various metrics to assess the effectiveness of the dimensionality reduction techniques combined with SVM classification. This was implemented by calculating a range of performance evaluation metrics including accuracy, precision, recall, and confusion matrix. These metrics were compared and analyzed with respect to ground truth labels to determine the performance of each technique in discriminating between different classes of hands based on HS images.

### 3.6. Visual Representation of Classification Result

For improved clarity and comprehensibility of the classification outcomes using PCA, FPCA, and WaLuMI techniques, a systematic approach was employed. The initial step involved a thorough analysis by varying the number of spectral bands to assess their impact on classification accuracy. This preliminary step was crucial in determining the optimal number of bands that would yield the most accurate results.

Subsequently, with the optimal number of bands identified, a visual representation of the classification result at the optimum was generated. This visual representation enhances the comprehension of classifier performance and effectiveness.

**Figure 8:**
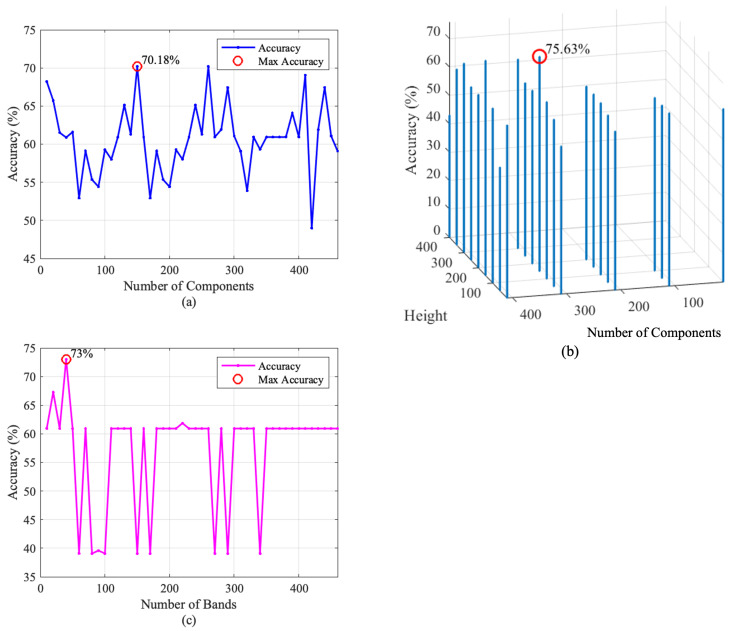
Accuracy plots for the three techniques evaluated on the right hand image using an increasing number of bands as features. Optimal points highlighted in red: (**a**) PCA (150 components), (**b**) FPCA (window size of 151×3, 310 components), and (**c**) WaLuMI (40 bands).

To offer a detailed perspective on this process, this process can be divided into two key steps:

1. *Assessing Optimal Number of Bands:* The classification accuracies were plotted against the varying number of bands as shown in Figure 8. This step allowed the identification of the point at which the classifier achieved its highest accuracy. The chosen number of bands at this point was regarded as the optimal configuration for subsequent analysis.
2. *Visual Classification Outcome:* With the optimal number of bands established, a visual representation of the classification outcome at the optimum was created. The produced image facilitates visual comparison with the ground truth. This provides insights into the performance of the classifier by illustrating the veins identified in the tested HS image (see figures in Section 4.2.1, Section 4.2.2, Section 4.2.3).

## 4. Results and Discussion

In this section, the results obtained from applying PCA, FPCA, and WaLuMI dimensionality reduction techniques are presented. The objective was to assess the effectiveness of these techniques and their performance in the context of vein detection using HS data.

### 4.1. Experiments and Results

To determine the optimal operating points of the three-dimensionality reduction methods, PCA, FPCA, and WaLuMI, 60% of the HS images of the dataset were randomly selected to train the SVM classifier, and the rest of the images were used to generate experimental results. The experimental procedures for each of the techniques are elaborated in the following subsections.

#### 4.1.1. PCA Experiments

The initial set of experiments applied PCA to the HS image data, systematically varying the number of principal components from 10 to 462 in steps of 10 to assess its impact on classification performance. As shown in Figure 8a, the experiments uncovered a complex interplay between the number of principal components and classification accuracy. While higher numbers of components often contributed to improved accuracy, it was observed that this trend did not hold uniformly across all ranges of component values. Instead, there were regions where increasing the number of components resulted in lower accuracy, indicating the presence of peak ranges for component selection. Beyond this range, further increases in components led to diminishing returns and, in some cases, decreased accuracy. From Figure 8a, it can be seen that the PCA-based method achieves its highest performance in terms of accuracy when it uses 150 components.

#### 4.1.2. FPCA Experiments

The second set of experiments delved into FPCA, which considers the window parameters (Height (H) and Width (W) of the window). The experiments aimed to understand the influence of both, the number of components and window parameters, on the classification accuracy using FPCA.

Figure 8b shows a three-dimensional plot representing the achieved accuracy versus the window’s height and the number of components. From Figure 8b, it is clear that the FPCA-based method achieves its optimum performance in terms of accuracy when it uses 310 components and a window size of 151×3.

Moreover, it is evident that the choice of the window parameters (H×W) significantly impacted the results. Smaller window height values often led to improved accuracy, particularly when dealing with a high number of components.

#### 4.1.3. WaLuMI Experiments

The third set of experiments focused on WaLuMI, specifically investigating the number of components and their influence on classification accuracy. Figure 8c demonstrates the accuracy versus the reduced number of bands for the WaLuMI-based method. From this figure, it can be observed the WaLuMI-based method achieves its highest performance in terms of accuracy when it reduces the dimensionality of the HS images to 40 bands.

Concerning the effect of dimensionality reduction, WaLuMI demonstrated competitive accuracy compared to PCA and FPCA. For instance, with 40 components, WaLuMI achieved an accuracy of approximately 73%.

The outcomes of these experiments provide valuable insights into the applicability of PCA, FPCA, and WaLuMI in the context of HS image classification for vein detection. Each of these techniques revealed distinct advantages, with FPCA particularly standing out by achieving the highest classification accuracy in the experiments. The selection of a method and its parameter configuration in the context of this study should be guided by the specific demands of the HS vein detection task. Considerations should encompass factors such as the dataset’s dimensionality and the distinctive spectral attributes of veins under investigation. These findings emphasize the necessity of aligning the choice of dimensionality reduction techniques with the intricacies of the vein detection challenge addressed in this research.

To generate experimental results, the calculated optimal operation parameters for PCA-, FPCA-, and WaLuMI-based methods were used to reduce the dimensionality of the input HS image data, where 60% of the input HS images of the dataset were used for training the SVM classifier and the rest of the images were used to generate the statistics. The obtained results for PCA, FPCA, and WaLuMI are presented in Table 3.

As shown in Table 3, in the evaluation of the three techniques, several key metrics were considered, including the accuracy, precision, recall, false positive rate (FPR), and false negative rate (FNR), which provide crucial insights into their classification performance.

PCA exhibited a relatively low FPR, suggesting that it had a commendable ability to correctly classify non-vein pixels without generating an excessive number of false alarms. However, a notable drawback is observed in its performance in terms of FNR. PCA exhibited a higher FNR, implying that it missed a considerable number of vein pixels during the classification process, leading to a significant number of false negatives. The overall accuracy of PCA is 70.18%, indicating that it successfully classified around 70.18% of the vein and non-vein pixels. The precision and recall values for PCA are 76.48% and 33.90%, illustrating its ability to balance between true positives and false positives.

FPCA demonstrates a slightly higher FPR compared to PCA, meaning that it has a relatively higher rate of false positives. This might lead to a slightly increased number of false alarms. However, FPCA excelled in capturing vein pixels, as indicated by its considerably lower FNR. The overall accuracy of FPCA is the highest among the three techniques, with a rate of 75.63%. This implies that FPCA correctly classified approximately 75.63% of the vein and non-vein pixels. The precision and recall values for FPCA are 73.34% and 59.12%, underlining its effectiveness in achieving both high true positives and low false positives.

Furthermore, WaLuMI shows a competitive FPR, striking a balance between classifying non-vein pixels correctly and avoiding false positives. Nonetheless, it has a higher FNR when compared to FPCA, signifying that it also missed some vein pixels during classification. The overall accuracy of WaLuMI is 73.00%, which means it successfully classified approximately 73.00% of the vein and non-vein pixels. The precision and recall values for WaLuMI are 78.03% and 43%, reflecting its ability to provide balanced classification results.

These results show that FPCA excelled in achieving the highest overall accuracy. Its strength lies in minimizing false negatives, even though it resulted in a slightly higher rate of false positives. PCA and WaLuMI demonstrated their own strengths and weaknesses, with PCA being effective at avoiding false positives and WaLuMI offering competitive accuracy. These findings highlight the importance of choosing dimensionality reduction techniques that fit the specific needs of the vein detection task, considering the trade-off between false positives and false negatives.

The dataset of 200 HS images of human hands, carefully curated, contributes significantly to the methodology’s reliability. The calculated optimal operation parameters for PCA, FPCA, and WaLuMI are used for dimensionality reduction, and the obtained results showcase the effectiveness of these techniques. The dataset’s diversity, covering a wide range of skin tones, enhances the generalizability of the vein detection algorithm. The ground truth annotations, crafted under the guidance of an expert, ensure accuracy and reliability in vein localization, further contributing to the robustness of the methodology. Overall, the comprehensive dataset plays a crucial role in training and evaluating the vein detection algorithms, reflecting real-world scenarios and contributing to the methodology’s reliability.

### 4.2. Morphological Operations

After performing vein detection using PCA, FPCA, and WaLuMI techniques, the obtained results were enhanced through morphological operations. This section presents the morphological operations applied for each dimensionality reduction technique.

#### 4.2.1. Morphological Operations for PCA

Morphological operations, including erosion and dilation, were strategically employed to extract and refine vein structures from the classified image for PCA. The morphological erosion involved the use of a disk-shaped structuring element with a radius of 4 pixels, iteratively reducing noise and filling gaps in the classified image. Additionally, an iterative dilation operation with a line-shaped structuring element (length: 5 pixels, angle: 180 degrees) was applied to enhance feature extraction.

**Figure 9:**
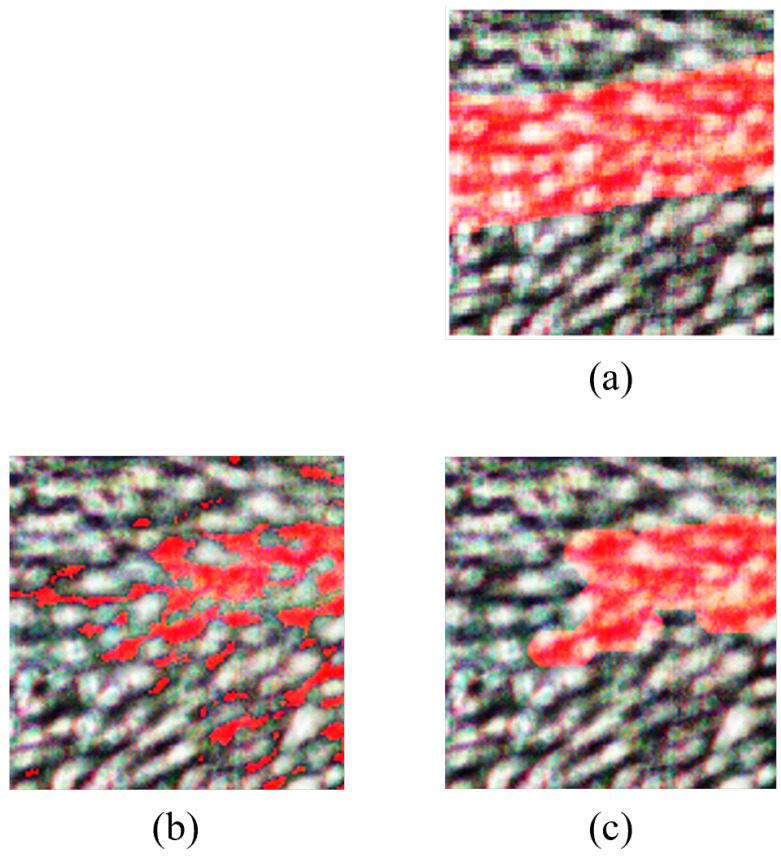
Results for PCA morphological operations. (**a**) RGB and ground truth overlay, (**b**) classified image, (**c**) refined image after morphological operations. Veins are highlighted in red.

From Figure 9, it can be seen that the PCA image exhibits relatively lower vein detection clarity. It indicates that PCA may not be the optimal choice for vein detection in HS images without further refinement.

#### 4.2.2. Morphological Operations for FPCA

For FPCA, iterative morphological erosion and dilation operations were applied to refine feature extraction. The morphological erosion involved an iterative process with a disk-shaped structuring element (radius: 4 pixels) to refine feature extraction. Dilation operations were then applied iteratively using a square-shaped structuring element (size: 2 × 2 pixels) to further enhance feature extraction.

**Figure 10:**
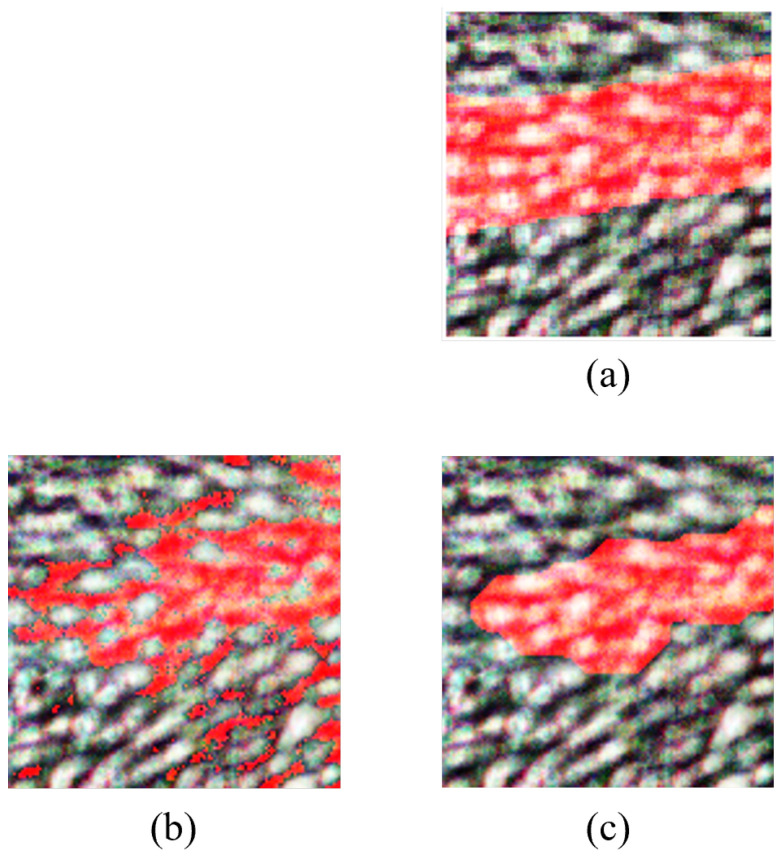
Results for FPCA morphological operations. (**a**) RGB and ground truth overlay, (**b**) classified image, (**c**) refined image after morphological operations. Veins are highlighted in red.

From Figure 10, it can be observed that the FPCA refined image has a vivid representation of vein structures, where vein regions in this image are prominently identified, demonstrating the high accuracy achieved by FPCA. The refined image further enhances the visualization, underscoring the method’s efficacy in isolating veins from the rest of the hand, making it a compelling choice for vein detection in HS images.

#### 4.2.3. Morphological Operations for WaLuMI

WaLuMI morphological operations involved the use of disk-shaped structuring elements with varying radii for morphological erosion and specific structuring elements for morphological dilation. The morphological erosion employed disk-shaped structuring elements with varying radii to iteratively reduce noise and gaps in the binary image. Dilation operations were then applied iteratively with structuring elements tailored to address specific characteristics of the data.

**Figure 11:**
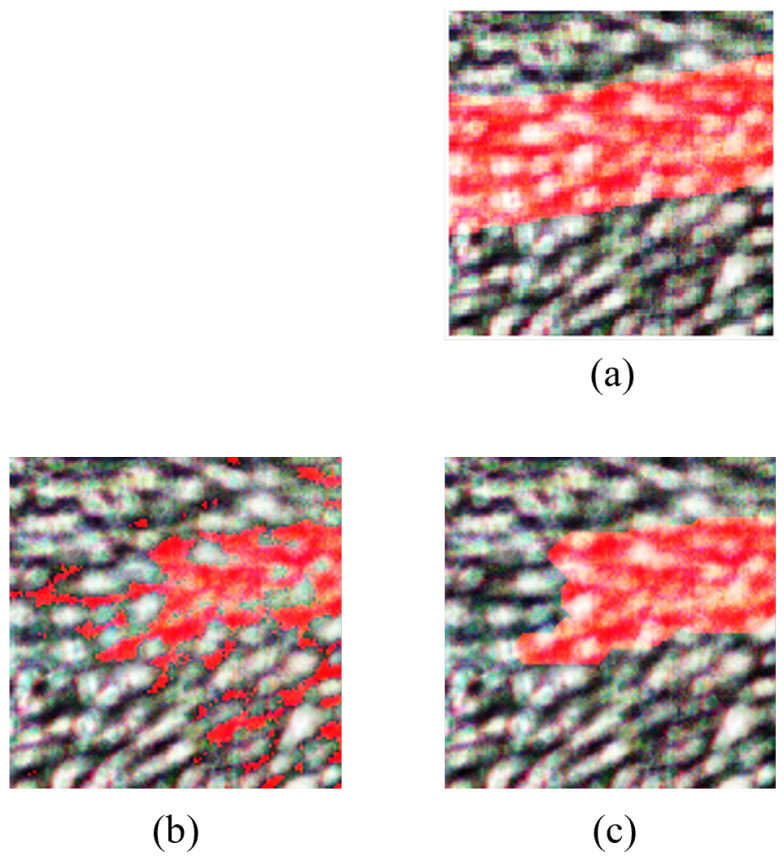
Results for WaLuMI morphological operations. (**a**) RGB and ground truth overlay, (**b**) classified image, (**c**) refined image after morphological operations. Veins are highlighted in red.

From Figure 11, it is evident that the refined image exhibits a notable degree of vein detection, though with slightly lower contrast compared to FPCA. Morphological operations enhance the image further, making it a viable choice for vein detection tasks, especially when factors such as computational efficiency are taken into account.

The outcomes of this visualization align with the quantitative results, where FPCA demonstrated the highest vein detection accuracy. Figure 8 illustrates how different dimensionality reduction techniques impact vein detection quality, emphasizing the importance of method selection based on the specific demands of the application. The remarkable visual results achieved with FPCA hold great promise for enhancing vein detection in various clinical contexts, paving the way for advancements in medical diagnostics and imaging.

## 5. Conclusions

In conclusion, this paper leveraged hyperspectral (HS) images to advance the field of vein detection, addressing the pressing need for improved diagnostic tools in various clinical settings. The curated dataset consisted of 100 subjects’ HS hand images with varying skin tones. To harness the potential of HS data for vein detection, three dimensionality reduction techniques, namely Principal Component Analysis (PCA), Folded Principal Component Analysis (FPCA), and Ward’s Linkage Strategy using Mutual Information (WaLuMI) were employed.

Through rigorous experimentation and evaluation, FPCA emerged as the standout performer, delivering the highest accuracy in vein detection. This result highlights the importance of optimizing dimensionality reduction methods in the pursuit of enhanced medical imaging and diagnostics.

Furthermore, the research extended beyond accurate classification to visualizing vein regions effectively. This was achieved by generating classified images using the optimal bands obtained from the dimensionality reduction techniques. These images were then refined through the application of morphological operations, providing clearer and more interpretable representations of vein structures.

The implications of this research are substantial, as it not only demonstrates the potential of HSI in conjunction with tailored dimensionality reduction techniques but also sets the stage for future investigations into advanced detection methods, including the incorporation of deep learning. The findings of this paper hold great promise, with the potential to significantly impact clinical practices and improve patient care in various healthcare settings.

The key contributions of this paper are as follows:

- Curated a diverse HS dataset with left- and right-hand captures from 100 subjects, addressing the need for varied skin tone representation.
- Explored three dimensionality reduction techniques (PCA, FPCA, WaLuMI) to optimize vein detection in HS images.
- Identified FPCA as the most effective technique, achieving the highest accuracy in vein detection.
- Extended the focus beyond accurate classification to include the effective visualization of vein regions.
- Generated classified images using optimal bands obtained from dimensionality reduction, refined through morphological operations for clearer representations.
- Demonstrated the potential of HSI with tailored dimensionality reduction, contributing significantly to medical imaging and diagnostics.

## Figures and Tables

**Table 1:** Summary statistics of the dataset.

Category	Count	Percentage
Total Participants	100	100%
Male	76	76%
Female	24	24%
**Ethnicity**		
African	32	32%
Asian	59	59%
European	9	9%
**Age Group**		
19–25	27	27%
26–30	28	28%
31–35	23	23%
36–40	20	20%
41–45	2	2%
**Skin Tone**		
Type I (Light)	15	15%
Type II (White)	19	19%
Type III (Medium)	21	21%
Type IV (Olive)	22	22%
Type V (Brown)	15	15%
Type VI (Black)	8	8%

**Table 2:** Summary statistics of the skin tones distribution of the dataset using ethnicity criteria [19].

Ethnicity	Skin Tone	Count	Percentage
European	Type I (Light)	6	6%
	Type II (White)	2	2%
	Type III (Medium)	1	1%
	Type IV (Olive)	0	0%
	Type V (Brown)	0	0%
	Type VI (Black)	0	0%
African	Type I (Light)	0	0%
	Type II (White)	0	0%
	Type III (Medium)	4	4%
	Type IV (Olive)	8	8%
	Type V (Brown)	13	13%
	Type VI (Black)	7	7%
Asian	Type I (Light)	9	9%
	Type II (White)	17	17%
	Type III (Medium)	16	16%
	Type IV (Olive)	14	14%
	Type V (Brown)	2	2%
	Type VI (Black)	1	1%

**Table 3:** Performance evaluation metrics for the three techniques on the HS image at their optimal parameters.

Method/Metric	Accuracy (%)	Precision (%)	Recall (%)	FPR (%)	FNR (%)
PCA	70.18	76.48	33.90	6.55	66.10
FPCA	75.63	73.34	59.12	13.78	40.88
WaLuMI	73.00	78.03	43.00	7.76	57.00

FPR = False Positive Rate, FNR = False Negative Rate.

## Data Availability

A subset of the dataset can be downloaded from: https://doi.org/10.5281/zenodo.10610238, accessed on 2 February 2024. The dataset is available for academic research upon request to the corresponding author.

## Abbreviations

The following abbreviations are used in this manuscript:

HS	HyperSpectral
HSI	HyperSpectral Imaging
PCA	Principal Component Analysis
FPCA	Folded Principal Component Analysis
WaLuMI	Ward’s Linkage Strategy using Mutual Information
CRPS	Complex Regional Pain Syndrome
NIR	Near-Infrared
ROI	Region Of Interest
SVM	Support Vector Machine
